# Indoor Mobility, Frailty, and Disability in Community-Dwelling Older Adults: A Mediation Model

**DOI:** 10.3390/ijerph191811386

**Published:** 2022-09-09

**Authors:** Paolo Riccardo Brustio, Anna Mulasso, Samuel D’Emanuele, Gianluca Zia, Luca Feletti, Susanna Del Signore, Alberto Rainoldi

**Affiliations:** 1 NeuroMuscularFunction, Research Group, School of Exercise & Sport Sciences, University of Torino, 10126 Torino, Italy; 2 Department of Neuroscience, Biomedicine and Movement, University of Verona, 37124 Verona, Italy; 3 Department of Clinical and Biological Sciences, University of Torino, 10126 Torino, Italy; 4 Department of Medical Sciences, University of Torino, 10126 Torino, Italy; 5 Bluecompanion Ltd., London NW8 9DD, UK; 6 Caretek s.r.l., 10127 Torino, Italy

**Keywords:** physical activity, walking, Adamo, physical frailty, ADL, active living, aged population

## Abstract

The general population, but especially older adults, were forced or encouraged to stay home during the recent COVID-19 pandemic. In this context, indoor mobility (IM, the number of steps performed daily at home) may be informative about the general health status of older adults. The present study aimed at evaluating the relationship between IM, frailty (loss of functional reserve including both physical and psychosocial domains), and disability (loss of autonomy measured as activities of daily life, ADLs) in a sample of community-dwelling Italian older adults. Specifically, the primary objective was to investigate IM and disability differences between robust and frail older adults. The secondary objective was to test if frailty is in the causal sequence between IM and disability, i.e., as a mediator in their relationship. Thirty-two participants (mean age = 70 ± 6 years; 56.2% women) were recruited. Frailty and disability were evaluated using the Tilburg Frailty Indicator and the Groningen Activity Restriction Scale, respectively. IM at home was measured via an Adamo wristwatch (a connected accelerometer). One-way analyses of covariance, controlling for age and gender, showed that robust participants, classified according to a score higher than five points in the Tilburg Frailty Indicator, performed significantly more IM (F_1,28_ = 4.639; *p* = 0.04) and presented lower disability grade than frail ones (F_1,28_ = 4.342; *p* =0.046). Only physical frailty was a mediator in the relationship between IM and disability (F_2,29_ = 8.538, *p* < 0.001), with a fully mediated model (z = −2.073, *p* < 0.04). Conversely, the total frailty score was not a mediator in the same relationship, but with IM accounted for the variance in disability (F_2,29_ = 8.538, *p* < 0.001; R^2^ = 33.7%). Our results suggested that frail older adults restricted their IM more and presented a higher level of disability compared to robust older adults. Moreover, data suggest that IM reduction may have a negative impact on physical frailty and indirectly increase disability.

## 1. Introduction

Elderly people aged 60 years and older are increasing in number and make up an increasing portion of the global population [1], implying severe consequences for healthcare and social services. With aging, a higher proportion of individuals is estimated to become frail [2,3]. The frailty status brings a higher risk for falls [4], cognitive decline [5,6], loss of autonomy [4], institutionalization [2,3], and hospitalization [4].

Regular physical activity is a key factor for healthy aging [7] and may reduce both mortality and common age-related diseases such as cardiovascular ones [7,8,9], type 2 diabetes [7,8,9], cancer [7,8,9], and depression [9]. Reducing physical inactivity during the aging process may help to preserve autonomy in activities of daily living (ADLs), prevent frailty onset [10], and improve health-related quality of life [10,11].

The American College of Sports Medicine, in collaboration with the American Heart Association [12], recommends 150 min/week of moderate-intensity physical activity in older adults for health benefits. More recently, a comment about the WHO 2020 Guidelines on Physical Activity and Sedentary Behaviour emphasized that inactive individuals should perform any amount of physical activity to reap their benefits, even when the recommended target range is perceived to be out of reach, and that overall, physical activity guidelines have shifted from exercise training to active living [13]. Nevertheless, most older adults do not meet the recommended physical activity level [14]. To encourage adherence to physical activity targets, Spiteri et al. [15] suggested acting on the main motivators of physical activity in older adults, such as social influences, reinforcement, and assistance in managing change. In Italy, older adults generally spend most of their daily time at home (more than 80%), and this trend increases with age [14]. For instance, an 85-year-old woman spends about 22 h and 27 min (94% of the day) at home. Similar data are found analyzing other European countries (e.g., Germany) [16]. Furthermore, frail older adults spend most of the day alone and inactive [17]. Therefore, in such a context, indoor mobility (IM) results in a more accessible form of physical activity and becomes the major contributor to physical activity [18].

In this regard, in recent years, Information and Communication Technologies (ICTs) have increased in popularity as a potential support to monitor older adults during everyday ADLs [19]. By using wearable or environment sensors, it is possible to quantify IM (e.g., number of steps), which is considered an important predictor of all-cause mortality and morbidity [20,21,22], cognition [23,24], risk of falling [25], frailty [26,27,28,29] and disability [30].

Therefore, assessing physical activity patterns in everyday life activity at home may allow us to characterize the relationship between activity level and age-related conditions, such as frailty. Even though the present study was conducted prior to the COVID-19 pandemic, the topic is of particular interest and has practical implications for the COVID-19 public health emergency. Indeed, it is worth pointing out that during the COVID-19 pandemic (representing an unprecedented challenge for the whole world), national authorities imposed restrictive measures to safeguard people’s health and the free movement of people was limited. As a result, older adults who are more vulnerable to COVID-19 were encouraged to stay at home throughout the day for many weeks or months. In this context, it becomes of interest to clarify if IM (i.e., the number of steps performed at home) can be informative about the general health status of older adults. While ICT tools have been applied to monitor physical activity levels in older adults, further research is needed to investigate how ICTs are used in frail older persons [19].

The present study aimed to evaluate the relationship between IM, frailty, and disability in a sample of community-dwelling Italian older adults. The main objective was to investigate IM and disability differences between robust and frail older adults. The secondary objective was to assess the role of frailty, as a mediator, in the relationship between IM and disability. Both components of frailty related to physical and psychosocial domains of human functioning were considered. We tested the hypothesis that robust and frail older adults have different patterns in terms of IM and ADLs. Specifically, we expected that robust older adults show a higher level of IM and score higher ADLs compared to frail ones. Specifically, we expected to observe a relationship between IM, frailty indexes (i.e., total, physical and psychosocial domains), and ADLs. Overall, we tested if older adults with a high level of IM are more prone to reporting fewer ADL limitations and less severe frailty, and finally if frailty mediates these effects.

## 2. Materials and Methods

### 2.1. Participants

Originally, thirty-five older adults agreed to participate in this observational study. The participants were recruited in two areas of Italy (Turin and Rome) from the local community via advertisements in University Centers. The data collection was performed from May to June 2017. Participants were eligible for the study if they were aged 65 years or over, living independently, and could understand the information provided during the testing. Participants were excluded if they were unable to walk independently with or without a walking aid or suffered from specific medical conditions, including acute diseases (e.g., recent fractures or surgical operation) and/or disabling illnesses that severely affect mobility (e.g., dialysis, respiratory insufficiency, coronary disease, known myocardiopathy, severe osteoarthritis). Participants were included if they presented chronic diseases that do not affect mobility (e.g., diabetes, hypertension). Three subjects were excluded because they did not meet the inclusion criteria.

Finally, thirty-two older adults (age 65–84 years; mean age = 70 ± 6 years), including 14 men (43.8%) and 18 women (56.2%), were enrolled in the study. Subjects were informed that their participation in the study was voluntary and confidential, and all participants provided written informed consent to participate in the study in accordance with the ethical standards of the Declaration of Helsinki (2016 update). The study protocol was approved by IMI (Innovative Medicine Initiative) in the grant agreement No 115,621 Sarcopenia and Physical fRailty IN older people: multi-componenT Treatment strategies—SPRINTT—and Amendment No 2, 22 May 2015. Caretek, a small and medium-sized enterprises company, was part of the project as the ICT provider.

### 2.2. Measures

Sociodemographic data were recorded, including age, gender, years of education (i.e., primary school, secondary school, high school diploma, university degree), family status (i.e., married, unmarried, widowed), chronic diseases (e.g., diabetes, hypertension, etc.), and pharmacotherapy using a self-report questionnaire. Frailty and disability were investigated through the Tilburg Frailty Index (TFI) and Groningen Activity Restriction Scale (GARS), respectively. To investigate IM at home, the participants wore a wristwatch accelerometer (Adamo) for a 7-day window of observation.

The TFI [31,32] was a self-report questionnaire composed of 15 items about the components of frailty related to physical, psychological, and social domains of human functioning. The physical domain consisted of eight questions on physical activity, unexplained weight loss, difficulty in walking, balance, vision problems, hearing problems, hand strength, and physical tiredness. The psychological domain comprised four questions about cognition, depressive symptoms, anxiety, and coping. The last three items belonged to the social domain and were related to living alone, social relations, and social support. The physical domain score (Physical TFI) ranged from 0 to 8 points, the sum of the psychological and social domains (Psychosocial TFI) ranged from 0 to 7 points, and the total TFI score ranged from 0 to 15 points given for each participant, with a higher score indicating greater frailty. Moreover, the sample was divided into frail and robust individuals using the cut-off of 5 points at the total TFI score.

The GARS [33] is a self-reporting questionnaire evaluating the disability both in basic ADLs (e.g., washing or dressing oneself) and in instrumental ADLs (e.g., shopping, preparing meals). It is composed of 18 items (11 items for basic ADLs, score range from 0 to 44 points; 7 items for instrumental ADLs, score range from 0 to 28 points) with a range from 18 to 72 points. A higher score indicates a higher likelihood of disability in ADLs. Using this cut-off, the sensitivity was good and specificity acceptable for most adverse outcomes, including disability, hospital admission, and receiving personal care, nursing, and informal care [34].

The Adamo system (designed by Caretek S.r.l., Torino, Italy) is a remote monitoring device for older adults, including a base station at the user’s home and a wristwatch accelerometer worn by the same user. The algorithm exploits the features of a 3-axis accelerometer (ADX346, Analog Devices, Norwood, MA, USA) sampling at 50 Hz [35]. The accelerometer is mounted on the watch so that when the arm is alongside the body, the x, y, and z axes are in the vertical, anterior–posterior, and mediolateral directions. The raw data were filtered and processed in real time via a dedicated firmware routine. For more explanation about the device, please see [35]. Every 10 min, a radio signal sent a data package to the base station.

The Adamo device accurately measured the number of steps at low walking speeds [35] typical of elderly participants and in a home environment, providing wider information about older adults’ health status [27]. Participants were instructed to wear the wristwatch for a 7-day period. The IM (i.e., number of steps performed at home) was calculated from data extracted from the Adamo web service.

### 2.3. Statistical Analysis

Descriptive analyses were carried out for every study variable. One-way analyses of covariance, with age and gender as covariates, were conducted to compare IM and GARS between robust and frail older adults. The thresholds for Partial eta squared were: >0.01, small effect size; >0.06 medium; >0.14 large effect size.

Pearson’s correlations were computed to assess the hypothesized relationships between the individual study variables: predictors (IM), mediators (TFI, Physical TFI, and Psychosocial TFI), and outcome (GARS). Threshold values for effect size statistics were: >0.1, small; >0.3, medium; >0.5, large effect size [36].

To prove our hypothesis, we performed a separate mediation analysis for the TFI or the Physical TFI. To test the relationship between IM and GARS through Physical TFI, a mediation analysis was performed according to the approach described by Baron and Kenny [37]. The mediation analysis was carried out by using multivariate regression analysis [38]. First, the direct effects of the IM on the GARS were determined by using regression analyses. Second, if the relationship was significant, the mediator was included in the model, and the main effect between the independent variable (IM) and the mediator (TFI or Physical TFI) was verified. Third, the mediation effect of the TFI or Physical TFI on the relationship between the independent variable (IM) and the outcome (GARS) was checked. Finally, the mediation model of the dependent variable was verified. The Sobel test was used to verify the mediation models. As the relationship among the IM, Psychosocial TFI, and GARS was not significant, no mediation analysis was performed. The Statistical Package for Social Sciences (SPSS 26.0 for Windows) and statistical package R (version 4.0.3; Foundation for Statistical Computing, 2018) were used for all statistical analyses. Significance levels were set at *p* ≤ 0.05 for all tests.

## 3. Results

Table 1 shows the details of the sociodemographic data of all participants. Most of the participants were married (59.4%) with a level of education corresponding to a high school diploma (43.8%). A relevant proportion of participants reported one or more chronic diseases (59.4%) and taking medicines regularly (68.8%). On average, participants presented an IM of 26,735 ± 12,752 steps per week, a TFI total score of 4 ± 2 points, a physical TFI score of 2 ± 2 points, a psychosocial TFI score of 2 ± 1 points, and a GARS score of 22 ± 5 points. Fifteen (46.9%) participants were categorized as frail (TFI score ≥ 5 points) and 17 (53.1%) as robust older adults. For more details, see Table 1.

Table 2 provides the results of IM and GARS scores separately for robust and frail older adults and the relevant analyses of covariance. Using the age and gender as covariates, the analyses of covariance showed significant differences for the robust and frail older adults both in IM (F_1,28_ = 4.639; *p* = 0.040; partial η^2^ = 0.142) and in GARS (F_1,28_ = 4.342; *p* = 0.046; partial η^2^ = 0.134). Specifically, IM was statistically significantly greater in robust compared to frail older adults (mean difference of 9921 (95%CI) (486, 19,356) steps). The GARS was lower in robust older adults than frail older adults (mean difference of −4 (−8, −0.1) points).

Moderate inverse correlations were observed between IM and GARS (r = −0.353, *p* = 0.047) and between IM and the TFI (r = −0.358, *p* = 0.044). IM negative correlated with the physical TFI (r = −0.429, *p* = 0.014) with a moderate effect size. A significantly larger direct correlation was observed between GARS and the TFI (r = 0.557, *p* = 0.001) and between GARS and the physical TFI (r = 0.599, *p* < 0.001). Differently, no significant correlation was observed between psychosocial TFI and IM (r = −0.151, *p* = 0.409) and GARS (r = −0.307, *p* = 0.087). For more details, see Table 3.

Considering the TFI as a mediator, the main effect of the mediator on the outcome (GARS) was statistically significant (β = 0.557, *p* = 0.001), as the main effect of the predictor (IM) on the outcome (GARS; β = −0.353, *p* = 0.047). Nevertheless, no mediation effect was observed (Sobel test: z = −1.725, *p* < 0.08). Despite this, TFI and IM account all together the 33.7% of the variance of GARS (F_2,29_ = 7.386, *p* = 0.003).

**Figure 1:**
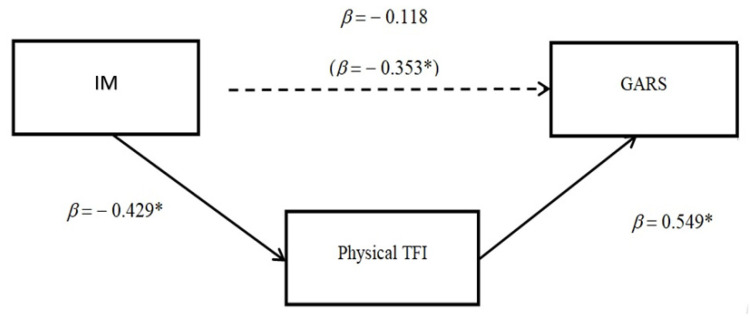
Mediation model among GARS (outcome), IM (predictor), and Physical TFI (mediator). Notes: Sobel test: z = −2.073, *p* < 0.04; * denotes *p* < 0.05.

Conversely, the mediation model results were significant when the Physical TFI was considered as a mediator of the relationship between IM and GARS. Specifically, before the introduction in the model of the mediator (i.e., Physical TFI), a negative relationship was observed between IM and GARS (β = −0.353, *p* = 0.047), and a positive one between the mediator and GARS (β = 0.599, *p* < 0.001). When Physical TFI was entered into the model as a mediator, the direct relationship between IM and GARS decreased (β = −0.118, *p* = 0.476). Nevertheless, mediation analysis revealed that there was a significant indirect effect of IM through the physical TFI on the changes in GARS. The goodness of fit (i.e., R^2^) explained 37.1% of the variance (F_2,29_ = 8.538, *p* < 0.001). The Sobel test indicated that the mediation model was fully mediated (z = −2.073, *p* < 0.04). For more details about the regression outcomes, please refer to Figure 1 and Supplementary Materials.

## 4. Discussion

The present study has examined three important variables acting on individual health (i.e., IM, frailty, and disability) and their relationships in a sample of Italian community-dwelling older adults. Firstly, we aimed to understand if frail and robust older adults showed different levels of IM measured using a connected accelerometer as the number of steps performed at home and if they differed for disability. Secondly, we tested if frailty, considering total frailty (first model) and the physical domain (second model), can mediate the relationship between IM and disability. If in the past, studies analyzing IM involved mainly older adults suffering from severe limitations contraindicating outdoor activities, our study focused on a sample of relatively healthy older adults who preserves the ability to walk independently and partial/total autonomy in the execution of ADLs.

Concerning the primary objective, as expected, the study revealed lower IM at home for frail compared to robust individuals. In frail older adults, a lower number of steps at home was performed weekly (~32%). This finding is consistent with other results showing a reduction of about 7% hourly physical activity for each frailty point measured using an adapted 4-item version of the frailty phenotype in a sample of US older adults from the National Social Life, Health and Aging Project [39] and a decrease of about 35% of light physical activity between most frail and robust people using a frailty index based on the deficit accumulation approach in a sample of people aged 50 and older from the National Health and Nutrition Examination Survey [40]. Yuki et al. [29] identified 5000 steps/day as the predictive cut-off for frailty development in Japanese older adults. Similarly, we found a different IM pattern between robust and frailty individuals. Indeed, using the TFI to discriminate between robust and frail we found that, on average, frail older adults performed about 3000 steps/day while robust ones took about 4500 steps/day. Evidence demonstrates that frail older adults are less likely to achieve the amount of physical activity suggested by the international guidelines than robust ones [40] and spend more time in sedentary activities [40,41,42,43]. Furthermore, breaks in sedentary time resulted negatively associated with frailty [41]. Our results assume even greater significance in the context of the COVID-19 pandemic, strengthening possibilities to effectively respond to potential persistent COVID-19 pandemic and/or to new public health emergencies characterized by social restrictions, i.e., lockdowns. In fact, it is acknowledged that older adults reported a substantial decrease of physical activity during the COVID-19 pandemic [44]. It was suggested that frailty can be reduced by replacing 30 min/day of sedentary behavior with moderate–vigorous physical activity [45]. Overall, the present study objectively confirmed that frail people are more disabled (~19%) than robust ones, as previously highlighted by other researchers both according the frailty phenotype [4] and the TFI [31].

Regarding the secondary objective, partially supporting our hypothesis, our study demonstrated that IM, frailty (total TFI score and physical TFI score), and disability are all related to each other with a moderate to large effect size. Furthermore, the social and phycological frailty domain appears unrelated to IM or disability. Even if we assessed disability by considering both basic and instrumental activities of daily living (i.e., GARS questionnaire) [46], it is worth pointing out that this scale primarily focused on physical health [47]. Indeed, in our results, physical frailty (i.e., physical TFI score) is a mediator between IM and disability. This means that IM influences physical frailty, which in turn influences autonomy. In other words, older adults with higher IM tended to be more autonomous in ADLs and the mediating role of physical frailty explains this effect. Additionally, we found that physical TFI score and IM account altogether about 37% of the variance in disability. Practically, our data underlined that specific physical interventions aimed to improve IM and mobility, might positively affect frailty and indirectly decrease disability. However, when we consider the total frailty score, the influence as a mediator was no longer found. Nevertheless, it is necessary to point out that the total TFI score and IM account altogether about 34% of the variance in disability. Close to our findings, a previous systematic review [45] showed a strong relationship between physical activity and disability; specifically, the risk of disability is reduced in physically active older adults (considering all types and intensity of physical activity) with an odds ratio of approximately 0.5. However, our study shows that just considering IM in independent older adults can be highly informative regarding disability. In line with the study of Miller et al. [48] on a sample of US older adults aged 70 and older, it is possible to suppose that physical activity can act on disability progression and possibly slow it. Furthermore, from a physiological point of view, a worsening of mobility status [44], frailty [49], and disability [50] are closely related to each other and associated with increased inflammatory activity. Specifically, high levels of IL-6, TNF-α, and CRP negatively influence mobility status and muscle strength [51], and, consequently, frailty. Frail and pre-frail people showed significantly higher levels of IL-6 and CRP compared to robust people [49]. The same inflammatory markers (i.e., IL-6 and CRP) that resulted are also associated with a loss of autonomy in ADLs [50].

Concerning potential mediators, it is interesting to note that Perrig-Chiello et al. [52] found that physical factors such as mobility can be strong predictors of autonomy in a large sample of older adults in the area of Basle. At the same time, the psychological resources contributed to the prediction of instrumental ADLs. In our study, functional autonomy was detected using the GARS questionnaire that comprises a significant number of basic ADLs items compared to instrumental ADLs ones (11 versus 7 items). This explains that frailty considered from a physical point of view influences the GARS as a mediator, while the total frailty (i.e., physical, and psychosocial domains) does not impact GARS as a mediator. Similarly, Gill et al. [53] found that physical frailty, measured according to the Fried phenotype, is a strong risk factor for disability onset, considering both progressive and catastrophic disability, in people aged 70 or older.

Some limitations should be considered in this study. First, the sample consisted of people motivated and interested in participating in the study. Thus, caution is needed to generalize these findings to other sets of older adults. Moreover, it is necessary to point out that the study was conducted in a specific cultural context (i.e., Italy); thus, this feature could have partially affected the study results. Indeed, it is possible that in other countries and cultures, the results could be different. Additionally, the nature of the study (i.e., cross-sectional design) does not allow us to discuss the relationship between the variables considered in the study and any possible causal inference. Finally, another limitation could be related to the seasonal variation that might have influenced our results. For example, environmental factors, such as weather or daylight hours, should be considered in interpreting the data.

## 5. Conclusions

It is possible to underline some practical implications deriving from our results: (i) monitoring IM levels in older adults may enable providing indirect information related to broader health parameters in autonomous older adults, such as frailty and autonomy, strictly linked to individual health status-related quality of life; and (ii) the need to promote health interventions supporting an active lifestyle (e.g., achievement of physical activity targets suggested by international guidelines) acting also on IM; (iii) the importance to differentiate and adapt health promotion interventions according to frailty status of older adults in a way that they become as effective and applicable as possible.

In summary, robust older adults presented higher levels of IM (i.e., number of steps performed at their home) than frail ones. Furthermore, IM can be a helpful parameter able to provide wider information on individual health status and is strictly related to frailty and disability in the aged population. The mediator role of physical frailty between IM and disability within this context is evident. Since the recent COVID-19 pandemic continues to impose social restrictions on the general population, and particularly in the case of frail older adults, to stay at home, it is worth considering the meaningful role that indoor activity may assume.

## Figures and Tables

**Table 1:** Sociodemographic data of the sample.

Variables	*n* (%)	Mean (SD)
Age, years	-	70 (6)
Gender, *n* (%) of female	18 (56.2)	-
BMI (kg/m^2^)	-	30 (7)
Marital status		-
Married	19 (59.4)	
Unmarried	3 (9.4)	
Widowed	10 (31.2)	
Level of education		-
Primary school, 5 years	5 (15.6)	
Secondary school, 8 years	9 (28.1)	
High school diploma, 13 years	14 (43.8)	
University degree, 18 years	4 (12.5)	
Chronic disease, *n* (%) of Yes	19 (59.4)	-
Pharmacotherapy, *n* (%) of Yes	22 (68.8)	-
TFI, points	-	4 (2)
Physical TFI, points	-	2 (2)
Psychosocial TFI, points	-	2 (1)
GARS, points	-	22 (5)
IM, steps per week	-	26,735 (12,752)

Notes: SD, standard deviation; BMI, Body Mass Index; TFI, Tilburg Frailty Index; GARS, Groningen Activity Restriction Scale; IM, Indoor Mobility.

**Table 2:** ANCOVA outcomes.

	Robust People Mean (SD)	Frail People Mean (SD)	ANCOVA
IM (steps)	31,466 (10,426)	21,374 (13,337)	F = 4.639, p = 0.040 partial η^2^ = 0.142
GARS (points)	21 (3)	25 (7)	F = 4.342, p = 0.046 partial η^2^ = 0.134

Notes: SD, standard deviation; GARS, Groningen Activity Restriction Scale; IM, Indoor Mobility.

**Table 3:** Correlation matrix (95% CI).

	IM	GARS	TFI	Physical TFI	Psychosocial TFI
IM	1	-	-	-	-
GARS	−0.353 * (−0.625,−0.005)	1	-	-	-
TFI	−0.358 * (−0.628,−0.011)	0.557 ** (0.259, 0.758)	1	-	-
Physical TFI	−0.429 * (−0.467,−0.218)	0.599 ** (0.317, 0.758)	0.832 ** (−0.681, 0.995)	1	-
Psychosocial TFI	−0.151 (−0.475, 0.209)	0.307 (−0.046, 0.593)	0.810 ** (−0.642, 0.903)	0.349 (−0.000, 0.622)	1

Notes: * denotes *p* < 0.05; ** denotes *p* < 0.01.

## Data Availability

The data presented in this study are available on request from the corresponding author.

## Supplementary Materials

The following supporting information can be downloaded at: https://www.mdpi.com/article/10.3390/ijerph191811386/s1.
