# Supplementary material for: Indoor Mobility, Frailty, and Disability in Community-Dwelling Older Adults: A Mediation Model
Source: Int J Environ Res Public Health. 2022 Sep 9;19(18):11386. doi: 10.3390/ijerph191811386 (PMC9517026; doi:10.3390/ijerph191811386)
Supplement: Supplementary file 1 [file ijerph-19-11386-s001.zip › ijerph-1810669-supplementary.pdf]

Supplementary material

	B (95% CI)	GARS RMSE	R^2	F	p
Model 1					
IM	-0.177 (-0.508,0.155)	4.40	0.33	7.386	0.002
TFI	0.494 (0.163, 0.825)				
Model 2					
IM	-0.118 (-0.451, 0.216)	4.29	0.37	8.538	0.001
TFI	0.549 (0.215, 0.882)				

Notes: IM, Indoor Mobility; TFI, Tilburg Frailty Index; GARS, Groningen Activity Restriction Scale
